# A novel behavioral paradigm to assess multisensory processing in mice

**DOI:** 10.3389/fnbeh.2014.00456

**Published:** 2015-01-12

**Authors:** Justin K. Siemann, Christopher L. Muller, Gary Bamberger, John D. Allison, Jeremy Veenstra-VanderWeele, Mark T. Wallace

**Affiliations:** ^1^Multisensory Research Laboratory, Neuroscience Program, Vanderbilt UniversityNashville, TN, USA; ^2^Neuroscience Program, Vanderbilt UniversityNashville, TN, USA; ^3^Computer Software Engineering Department, MED Associates Inc.St. Albans, VT, USA; ^4^Murine Neurobehavior Core, Vanderbilt UniversityNashville, TN, USA; ^5^Center for Autism and the Developing Brain, and Department of Psychiatry, Sackler Institute for Developmental Psychobiology, Columbia UniversityNew York, NY, USA; ^6^Department of Hearing and Speech Sciences, Vanderbilt UniversityNashville, TN, USA; ^7^Department of Psychology, Vanderbilt UniversityNashville, TN, USA; ^8^Department of Psychiatry, Vanderbilt UniversityNashville, TN, USA

**Keywords:** multisensory integration, mouse behavior, operant conditioning, visual processing, auditory processing, mouse models

## Abstract

Human psychophysical and animal behavioral studies have illustrated the benefits that can be conferred from having information available from multiple senses. Given the central role of multisensory integration for perceptual and cognitive function, it is important to design behavioral paradigms for animal models to provide mechanistic insights into the neural bases of these multisensory processes. Prior studies have focused on large mammals, yet the mouse offers a host of advantages, most importantly the wealth of available genetic manipulations relevant to human disease. To begin to employ this model species for multisensory research it is necessary to first establish and validate a robust behavioral assay for the mouse. Two common mouse strains (C57BL/6J and 129S6/SvEv) were first trained to respond to unisensory (visual and auditory) stimuli separately. Once trained, performance with paired audiovisual stimuli was then examined with a focus on response accuracy and behavioral gain. Stimulus durations varied from 50 ms to 1 s in order to modulate the effectiveness of the stimuli and to determine if the well-established “principle of inverse effectiveness” held in this model. Response accuracy in the multisensory condition was greater than for either unisensory condition for all stimulus durations, with significant gains observed at the 300 ms and 100 ms durations. Main effects of stimulus duration, stimulus modality and a significant interaction between these factors were observed. The greatest behavioral gain was seen for the 100 ms duration condition, with a trend observed that as the stimuli became less effective, larger behavioral gains were observed upon their pairing (i.e., inverse effectiveness). These results are the first to validate the mouse as a species that shows demonstrable behavioral facilitations under multisensory conditions and provides a platform for future mechanistically directed studies to examine the neural bases of multisensory integration.

## Introduction

We live in a world comprised of a multitude of competing stimuli delivered through a number of different sensory modalities. The appropriate filtering, segregation and integration of this information is integral to properly navigate through the world and for creating a unified perceptual representation. Having information available from multiple sensory modalities often results in substantial behavioral and perceptual benefits (Stein and Meredith, 1993; Murray and Wallace, 2011; Stein, 2012). For example, it has been shown that in noisy environments, seeing and hearing an individual speak can greatly enhance speech perception and comprehension relative to just the audible signal alone (Sumby and Pollack, 1954). In addition, responses have been shown to be both faster and more accurate under combined modality circumstances (Calvert et al., 2004; Stevenson et al., 2014a). Numerous other examples of such multisensory-mediated benefits have been established (Calvert and Thesen, 2004; Stein and Stanford, 2008), and serve to reinforce the utility of multisensory processing in facilitating behavioral responses and in constructing our perceptual view of the world. Furthermore, emerging evidence points to altered multisensory processing in a number of human clinical conditions, including autism and schizophrenia, reinforcing the importance of having a better mechanistic understanding of multisensory function (Foss-Feig et al., 2010; Kwakye et al., 2011; Marco et al., 2011; Cascio et al., 2012; Martin et al., 2013; Stevenson et al., 2014b).

Numerous animal model and human imaging studies have explored the neural mechanisms that underpin multisensory processing (Stein and Meredith, 1993; Calvert and Thesen, 2004; Calvert et al., 2004; Stein and Stanford, 2008; Murray and Wallace, 2011; Stein, 2012; Stevenson et al., 2014a). These studies have highlighted the neural operations performed by individual multisensory neurons and networks, demonstrating the importance of stimulus properties such as space, time and effectiveness in determining the final product of a multisensory pairing (Meredith and Stein, 1985, 1986a,b; Meredith et al., 1987; Wallace and Stein, 2007; Royal et al., 2009; Stevenson et al., 2012; Ghose and Wallace, 2014). In addition, an increasing emphasis is now being placed on detailing how neuronal responses relate to behavioral outcomes under multisensory circumstances (Wilkinson et al., 1996; Stein et al., 2002; Burnett et al., 2004; Hirokawa et al., 2008).

Historically, these multisensory studies have focused on large mammalian models such as the cat and monkey, given the similarities in their sensory systems to humans and the ease with which both neural responses and behavior can be measured. With the advent of molecular genetic manipulations in mouse models and their applicability to human disease, however, there is a growing need to better understand sensory and multisensory function in these lower mammals. As highlighted above, this has become very germane of late as evidence grows for the presence of sensory and multisensory deficits in clinical disorders (Iarocci and Mcdonald, 2006; Kern et al., 2007; Smith and Bennetto, 2007; Cascio, 2010; Keane et al., 2010; Russo et al., 2010; Marco et al., 2011; Brandwein et al., 2012; Foxe et al., 2013; Stevenson et al., 2013, 2014c; Wallace and Stevenson, 2014).

In addition to molecular genetic manipulations such as knock-ins or knock-outs of genes associated with human disease, the rodent offers additional practical advantages spurred by the development of optogenetic methods to study causal relations in brain circuits (Fenno et al., 2011; McDevitt et al., 2014). Application of such tools to multisensory questions could be of great utility in developing a better mechanistic understanding of how the integrative features of multisensory neurons and networks arise, and how neuronal and network properties relate to behavior.

For these reasons (and others), recent studies have begun to focus on examining multisensory processes in rodent models (Sakata et al., 2004; Gleiss and Kayser, 2012; Raposo et al., 2012; Carandini and Churchland, 2013; Olcese et al., 2013; Sheppard et al., 2013; Sieben et al., 2013; Gogolla et al., 2014; Hishida et al., 2014). For practical reasons, this work has initially focused on the rat, and has established strong neural-behavioral links in this species (Tees, 1999; Komura et al., 2005; Hirokawa et al., 2008, 2011). However, the mouse remains the primary model for molecular genetic and optogenetic manipulations, where limited knowledge concerning multisensory function still exists.

The current study represents the first of its kind to systematically examine unisensory (i.e., auditory alone, visual alone) and multisensory (i.e., paired audiovisual) behavioral function in mice. The core objective of these experiments was to determine if multisensory processing is conserved in the mouse and similar to the features observed in larger animal models. Our ultimate objective is that this behavioral paradigm, in conjunction with neurophysiological methods, could then be used to detail the neural bases of multisensory function. The establishment of such links would then allow for the application of powerful genetic, pharmacologic and optogenetic tools to evaluate questions of mechanistic relevance. Finally, further studies may assess multisensory processing in mouse models of disease/disorder along with determining the underlying systems that may be atypical under these multisensory conditions.

## Materials and methods

### Animals

Nine male mice on C57BL/6J and 129S6/SvEv inbred strains were obtained from the Jackson Laboratory (Bar Harbor, ME, USA) and Taconic (Hudson, NY, USA), respectively. All animals were 4 weeks of age and housed in the Vanderbilt Murine Neurobehavioral Core with one cage mate. Mice were on a 12-h light/day cycle and given water *ad libitum*. For the first week, at 5 weeks of age, mice were given food *ad libitum* and handled daily to acclimate to the experimenters and facility. Since the proposed behavioral task requires mice to make a decision in order to obtain a food reward, animals were placed on a food-restricted diet. Mice were only given food *ad libitum* on weekends (non-testing days) and free access to food for 4 h every weekday, and this food restriction was gradually reached over a 2-week period before behavioral training began. Liquid vanilla Ensure (Abbott Laboratories, Abbott Park, IL, USA) was given in home cages for 60 min for 2 days before operant training began to expose animals to the reward. Body weights were recorded weekly and if an animal lost 20% of its initial weight, it would be excluded from the study until it had regained enough weight to participate based on this criterion. All experiments and protocols were approved by the Institutional Animal Care and Use Committee at Vanderbilt University.

### Equipment

**Figure 1:**
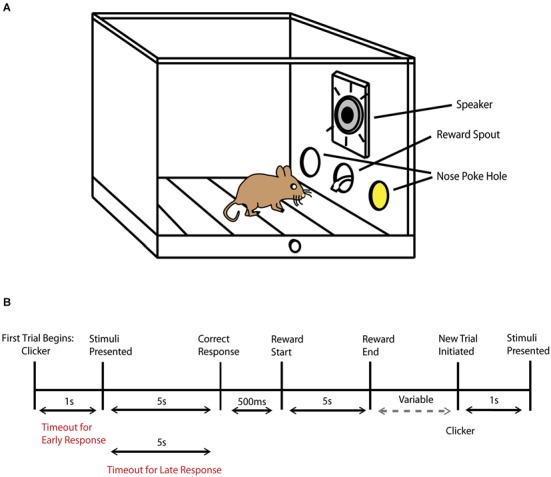
**Behavioral task schematic. (A)** Diagram of operant chamber during the presentation of an audiovisual stimulus (represented by the yellow color within the nose poke hole, where the LED was positioned) and by the active speaker. **(B)** Schematic representation of the trial sequence and timing. The phrase variable represents the amount of time that progresses until the animal decides to initiate a new trial by then performing a nose poke in the central hole.

Mice were placed in adapted mouse operant chambers (Med Associates Inc, St. Albans, VT, USA) that measured 7.0” L × 6.0” W × 7.25” H and were contained in sound attenuating cubicles. The chamber contained three nose poke holes with infrared sensors on the front wall, a house light, fan and clicker positioned on the rear wall and a mounted camera placed on the ceiling of the cubicle above the chamber. A section of metal mesh replaced the standard chamber plate and was placed above the central hole with a 3” × 3” horn tweeter (Pyle Pro Audio, Brooklyn, NY, USA) that was located behind the mesh section (Figure 1A). LED lights were contained within the left and right nose poke holes and emitted a standard intensity of 1 lux, characteristic of the operant chamber. To minimize the small possibility that outside light may enter into the chambers, training and testing experiments were conducted in dim red light. Auditory stimuli were comprised of either white noise or an 8 kHz tone played at 85 db SPL, which were measured and calibrated using a SoundTrack LxT2 sound level meter (Larson Davis, Provo, UT, USA). Non-significant sound level measurements were observed in each chamber as auditory stimuli were played in the remaining chambers to ensure that sounds from one operant chamber could not be heard in another chamber. All rewards were presented in the central nose poke hole and comprised of 0.1 cc of liquid vanilla Ensure dispensed by an automated dipper.

### Behavioral task

#### Initial training

On the first day of training mice were acclimated to the operant chamber. A reward was given in the central nose poke hole every 60 s for 1 h to demonstrate the location where the reward would be dispensed. In the next step of training, only the right nose poke hole was active. Mice were trained to respond in the right nose poke hole, which then resulted in a 500 ms visual stimulus presentation in the same location immediately followed by an Ensure reward. Once the number of responses was greater for the right vs. left nose poke hole for two consecutive days; the left nose poke hole was then activated and the right was inactivated. The initial training and reversal each lasted 4–5 days to meet the above criterion. This phase demonstrated that responding to either nose poke hole could result in a reward and was an early exposure to a sensory stimulus being paired with a reward.

#### Unisensory training

In the next step of the behavioral task, mice were presented with a visual light stimulus in either the left or right nose poke hole. The animal had 5 s to make a decision once the stimulus was presented, and a correct trial occurred when the animal responded to the nose poke hole where the visual stimulus was presented. A correct response resulted in an additional 5 s time period for the animal to collect the reward. In this training phase, mice also learned to initiate a new trial by nose poking in the central hole. After a correct response and a reward was obtained, there was a 2 s “wait” period where any response would not result in the start of a new trial, in order to minimize accidental or impulsive responses. After 2 s had passed, mice could then initiate a new trial at any time by nose poking in the central hole. Once a trial was initiated, a clicker (50 ms duration) would signal that a stimulus would be presented shortly, and, after a 1 s delay, a light stimulus appeared again either in the left or right nose poke hole in a pseudorandom order. Every incorrect trial resulted in a timeout, with the house light illuminating the chamber for 5 s, and no further responses could be made during this time period. In addition to timeouts for incorrect responses, timeouts could occur if the animal responded too early, before a stimulus was presented (<1 s), and if an animal waited too long (>5 s) before making a response (Figure 1B). As the mice became more accurate, the duration of the light stimulus was gradually reduced from 4 s to 2 s. For each training session mice completed a total of 100 trials (50 per side) for up to 90 min. Once the visual task was completed successfully for two consecutive days using a 65% correct response rate criterion, mice progressed to the auditory component of the task. In the auditory task, either white noise or an 8 kHz tone at a constant 85 db was played individually. Mice were trained to associate the tone with responding to the left nose poke hole and white noise with responding to the right nose poke hole. The trial description, behavioral sessions and criterion to advance to the next stage of the paradigm were the same as described in the visual component of the task.

#### Multisensory testing

After the visual and auditory training components of the task were completed, mice advanced to the behavioral testing phase where multisensory processing was evaluated. For multisensory trials only congruent/paired audiovisual trials were presented. Based on the variability of stimulus duration presentations in the multisensory rodent literature, we pragmatically selected a variety of stimulus durations. Unisensory and multisensory processing was evaluated for 5 days at each of the selected durations and proceeded by gradually shortening the durations. Therefore, mice were initially tested on 1 s presentations of visual alone, auditory alone and paired audiovisual stimuli for 5 days, and this was then evaluated at 300 ms, 100 ms and 50 ms stimulus durations. In these behavioral sessions, mice completed 150 trials (50 per condition presented in a pseudorandom order) lasting up to 90 min per testing day.

### Data analysis

All behavioral experiments were created utilizing customized Med-PC IV programs (Med Associates Inc.). Behavioral accuracies in the initial training phase were calculated by comparing the responses to the left vs. right nose poke hole (and vice versa during reversal learning) using two-tailed *t* tests. Accuracies measured for visual and auditory training sessions were calculated as percent correct utilizing a 65% correct response rate for two consecutive days to progress to the multisensory testing phase. Percent gain was calculated as (mean number of correct multisensory trials − mean number of correct best unisensory trials) / (mean number of correct best unisensory trials) × 100. Accuracy data was calculated as correct trials / correct + incorrect trials (misses only). Prism 6 (Graphpad Software Inc, La Jolla, CA, USA) was used to calculate all statistical analyses. Two-way analysis of variances (ANOVA) with repeated measures and Tukey’s multiple comparisons tests were utilized for all experiments unless otherwise specified. Mean and standard error of the mean is presented.

## Results

### Behavioral performance for unisensory (visual alone, auditory alone) training

**Figure 2:**
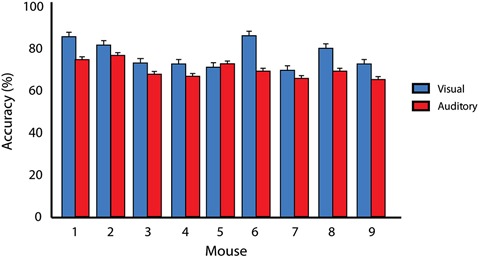
**Criterion performance on unisensory tasks**. Average behavioral accuracies for visual only (blue) and auditory only (red) training conditions for two consecutive days once animals had reached 65% correct criterion performance. A significant difference (*p* = 0.0002) in behavioral performance was found when comparing visual and auditory performance across animals. Error bars represent SEM.

Mice were trained to identify both visual and auditory stimuli on separate and independent tasks. Each training session for these unisensory tasks consisted of 100 trials that occurred once daily. Criterion performance occurred when mice achieved 65% correct performance for two consecutive days. Mice first completed the visual training component of the behavioral task. Once animals reached criterion, they then advanced to the auditory training component. Using this criterion, mice completed the visual task with a final accuracy of 77.3% ± 1.8% and completed the auditory task with a final accuracy of 70.1% ± 1.1%. A paired *t* test revealed significant differences between visual and auditory unisensory behavioral performance upon achievement of criterion (*p* = 0.0002; Figure 2). Substantial differences were noted in the time it took the mice to learn the two unisensory tasks. Whereas mice completed the visual task in 12.5 ± 0.95 days, it took 35.1 ± 4.55 days to complete the auditory task. Finally, unpaired *t* tests revealed no significant differences between mouse strains for behavioral accuracies for either visual training (*p* = 0.62) or for auditory training (*p* = 0.29).

### Unisensory and multisensory behavioral performance as a function of stimulus duration

**Figure 3:**
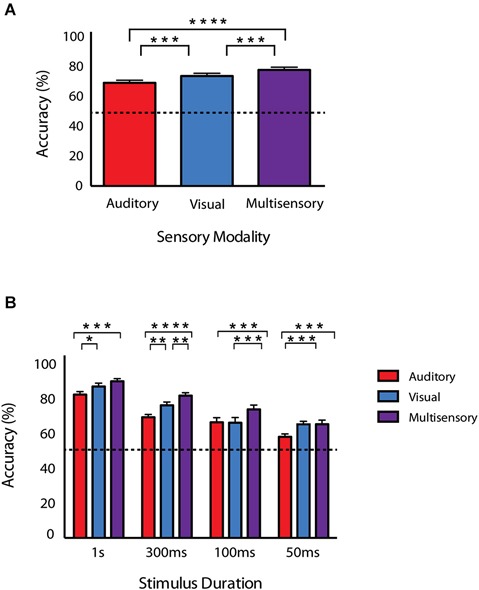
**Behavioral accuracy for auditory, visual and audiovisual conditions across stimulus durations. (A)** Accuracy for each of the conditions collapsed across all stimulus durations. **(B)** Accuracies as a function of sensory modality and duration. Note that response accuracy was greatest for multisensory compared to unisensory conditions across all of the tested durations. Data are presented from nine male mice of both C57BL/6J and 129S6/SvEv strains. Dotted line represents 50% accuracy or chance level. Error bars represent SEM. The significant levels are as follows: (* = *p* < 0.05, ** = *p* < 0.01, *** = *p* < 0.001, **** = *p* < 0.0001).

Once animals had achieved criterion performance for each of the unisensory tasks, they then performed a paired audiovisual version of the task. Furthermore, in order to modulate the effectiveness of the unisensory stimuli, the duration was varied. Mice were initially tested on the longest duration condition (i.e., 1 s) in response to visual, auditory and multisensory stimuli for 5 days. Following this, performance was then evaluated at durations of 300 ms, 100 ms and 50 ms. Collapsing across all durations, behavioral accuracies under multisensory conditions were greater than for visual or auditory only conditions (Figure 3A). Overall accuracy for these collapsed conditions was as follows: multisensory—77.8% ± 1.83, visual—73.7% ± 1.82 and auditory 69.1% ± 1.75. A repeated measures one-way ANOVA was used to evaluate accuracy as a function of sensory modality and revealed a significant difference (*p* < 0.0001; *F*_(1.907,66.75)_ = 39.88). Using Sidak’s multiple comparison *post hoc* test, we found significant differences between the multisensory and visual conditions (*p* < 0.001), multisensory and auditory conditions (*p* < 0.0001) and the visual and auditory conditions (*p* < 0.001). Next, a repeated measures two-way ANOVA with a Tukey’s multiple comparisons *post hoc* test was used to compare response accuracy for unisensory and multisensory conditions across the different stimulus durations (Figure 3B). Main effects of stimulus duration (*p* < 0.0001; *F*_(3,32)_ = 31.75), sensory modality (*p* < 0.0001; *F*_(2,64)_ = 46.65) and a significant stimulus duration × sensory modality interaction effect (*p* = 0.0125; *F*_(6,64)_ = 2.981) were observed. Table 1 shows response accuracy for each sensory modality and duration. When examined on a duration-by-duration basis, response accuracies under multisensory conditions were consistently greater than under either unisensory condition.

**Table 1:** **Behavioral accuracies for each sensory modality across stimulus durations**.

	Sensory Modality		
Stimulus duration	Multisensory	Visual	Auditory
1 s	90.0 (1.56)	87.5 (1.94)	82.3 (1.79)
300 ms	81.8 (1.60)	76.2 (1.92)	69.4 (1.58)
100 ms	73.9 (2.52)	66.2 (3.00)	66.5 (2.67)
50 ms	65.4 (2.33)	65.3 (1.70)	58.1 (1.63)

*Accuracies were greatest under multisensory conditions compared to unisensory conditions for all of the tested stimulus durations. Overall, accuracies decreased as the stimulus duration shortened. Data are presented from nine male mice of both C57BL/6J and 129S6/SvEv strains. SEM is represented in parentheses*.

We were also interested in assessing any effects of inbred strain. Using a repeated measures two-way ANOVA a main effect of sensory modality was observed at every stimulus duration across both strains (1 s; *p* = 0.004, 300 ms; *p* = 0.001, 100 ms; *p* = 0.001, 50 ms; *p* = 0.006). However, no significant main effect of mouse strain was observed at any stimulus duration (1 s; *p* = 0.084, 300 ms; *p* = 0.29, 100 ms; *p* = 0.97, 50 ms; *p* = 0.061).

### Evaluating multisensory gain

**Figure 4:**
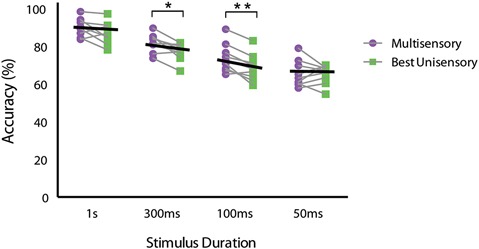
**Performance differences between multisensory and best unisensory conditions for individual animals**. Multisensory and the best unisensory performance accuracies were averaged separately for each mouse across the 5 days of testing for each stimulus duration. Black lines represent the group average performance under multisensory and the best unisensory conditions. Note the descending slope of these lines, which is most apparent for the 300 ms and 100 ms duration conditions. Data are presented from nine male mice of both C57BL/6J and 129S6/SvEv strains. The significant levels are as follows: (* = *p* < 0.05, ** = *p* < 0.01).

In order to measure the degree of facilitation attributable to having information available from both senses, we calculated multisensory gain by utilizing the equation (average multisensory correct trials − average best unisensory correct trials)/(average best unisensory correct trials) × 100 (Meredith and Stein, 1983). The greatest multisensory gain was seen for the 100 ms duration stimuli, with animals exhibiting a greater than 11% gain in performance. Using this calculation, we found multisensory gain at each of the tested stimulus durations (average gain at 1 s = 3.40%, 300 ms = 7.40%, 100 ms = 11.15%, and 50 ms = 0.10%). A similar pattern of gain was found for both mouse strains. To further evaluate multisensory gain, we then compared the original behavioral performance data for the multisensory and the best unisensory conditions for each individual mouse across all stimulus durations. A repeated measures two-way ANOVA with factors of stimulus duration and sensory modality with a Sidak’s multiple comparisons *post hoc* test was used. Significant main effects of stimulus duration (*p* < 0.0001; *F*_(3,24)_ = 40.1) and sensory modality were observed (*p* = 0.0073; *F*_(1,8)_ = 12.72), but no significant stimulus duration × sensory modality interaction (*p* = 0.068; *F*_(3,24)_ = 2.70) was observed (Figure 4). Utilizing the Sidak’s multiple comparison *post hoc* test, we found significant differences between the multisensory and best unisensory conditions at the 300 ms (*p* < 0.05) and the 100 ms stimulus condition (*p* < 0.01). Overall, multisensory gain was found to gradually increase as stimulus duration was shortened, with gain increasing up to a maximum at stimulus durations of 100 ms. Multisensory gain was observed to be significantly different from zero at the 300 ms and 100 ms conditions. Somewhat surprisingly, however, little gain was seen at the shortest duration (i.e., 50 ms), even though animals were performing above chance levels on unisensory trials. One possible explanation for this lack of effect at this shortest duration is the mismatch in performance between the visual and auditory trials. In a Bayesian framework, differences in performance between the two unisensory conditions would be expected to yield little gain because of the differences in reliability of the different sensory channels (here with vision being more reliable) (Deneve and Pouget, 2004; Beierholm et al., 2007; Murray and Wallace, 2011).

### Effects of testing day

**Figure 5:**
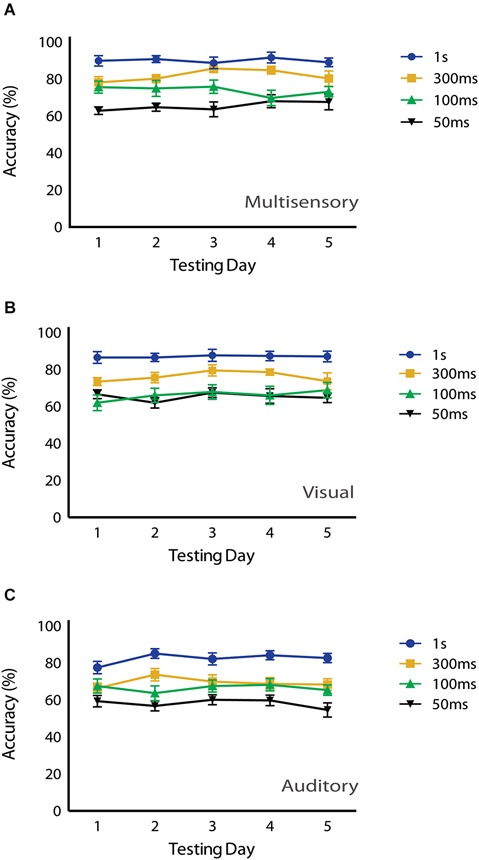
**Effects of testing day on behavioral performance**. No significant main effects of testing day were observed under **(A)** multisensory (*p* = 0.846), **(B)** visual (*p* = 0.381) or **(C)** auditory conditions (*p* = 0.514) using a repeated measures 2-way ANOVA (Tukey’s test). Data are presented from nine male mice of both C57BL/6J and 129S6/SvEv strains. Error bars represent SEM.

Lastly, in an attempt to determine if the potential novelty of the multisensory stimuli had any effect on behavioral performance, we evaluated accuracy as a function of testing day. Repeated measures two-way ANOVAs with Tukey’s multiple comparisons *post hoc* tests were used to compare behavioral accuracy for multisensory, visual and auditory conditions across testing days (Figure 5). For multisensory conditions, a significant main effect of stimulus duration (*p* = <0.0001; *F*_(3,32)_ = 26.65) was observed, but neither a significant main effect of testing day (*p* = 0.846; *F*_(4,128)_ = 0.346) nor a significant interaction effect (*p* = 0.465; *F*_(12,128)_ = 0.987) was observed. This pattern was also found for both the visual and auditory conditions. For the visual condition a significant main effect of stimulus duration (*p* = <0.0001; *F*_(3,32)_ = 21.49) was observed, yet neither a significant main effect of testing day (*p* = 0.381; *F*_(4,128)_ = 1.056) nor a significant interaction effect (*p* = 0.901; *F*_(12,128)_ = 0.502) was observed. Finally, for the auditory condition a significant main effect of stimulus duration (*p* = <0.0001; *F*_(3,32)_ = 26.03) was found, but neither a significant main effect of testing day (*p* = 0.514; *F*_(4,128)_ = 0.822) nor a significant interaction effect (*p* = 0.619; *F*_(12,128)_ = 0.831) was observed. Overall, we found that accuracies were consistent across the testing days, and there were no differences in performance levels for visual, auditory or audiovisual stimuli across the days of testing.

## Discussion

The current study is the first to evaluate behavioral performance under multisensory conditions in the mouse. A variety of studies have evaluated various facets of either visual or auditory behavioral function in mice (Pinto and Enroth-Cugell, 2000; Prusky et al., 2000; Klink and Klump, 2004; Radziwon et al., 2009; Busse et al., 2011; Jaramillo and Zador, 2014), yet none have focused on determining the behavioral effects when these stimuli are combined. Overall, we found that mice were more accurate at identifying paired audiovisual stimuli compared to either visual or auditory stimuli alone across all of the tested stimulus durations, with significant gains observed at the 300 ms and 100 ms durations. As a general rule, behavioral accuracy decreased as the stimulus duration was shortened down to the 100 ms duration, after which we believe that the stimulus duration was sufficiently short to be close to threshold detection levels. We suggest that this duration effect is in accordance with inverse effectiveness, a key concept in the multisensory literature, and complements a host of similar findings in human, monkey, cat, and rat model systems (Meredith and Stein, 1986b; Cappe et al., 2010; Murray and Wallace, 2011; Ohshiro et al., 2011; Gleiss and Kayser, 2012). Inverse effectiveness states that as the effectiveness of the unisensory stimuli decreases, greater behavioral benefits can be observed when these stimuli are combined compared to when the individual (visual or auditory) stimulus is presented alone (Meredith and Stein, 1986b). Although effectiveness in the larger animal models has been typically manipulated via changes in stimulus intensity, in our current study we were able to show corresponding effects in the mouse through manipulations of stimulus duration.

One key limitation from the current study is that we only manipulated one dimension of the sensory stimuli (i.e., duration). In the rat, stimulus intensity has been modulated to examine multisensory function (Gleiss and Kayser, 2012), and we hope to extend our studies into the domain of intensity in the future. Furthermore, due to practical constraints associated with the operant chambers, auditory stimuli were delivered from a single (central) spatial location, thus placing the visual and auditory stimuli somewhat out of spatial correspondence. Future work will add a second spatially congruent speaker to the operant chamber. These optimizations of the stimulus structure will likely reveal even larger multisensory interactions than those revealed in the current study, which we believe to be a conservative estimate of the potential gains in performance. However, we believe that the differences in multisensory gain by using either one or two speakers for this specific task would be minimal. The reasoning is that in this task we used a fairly loud auditory stimulus (85 db) from a centrally located speaker in a standard operant chamber that is 7.0” L × 6.0” W × 7.25” H, thus making the stimulus highly effective. Future work will indeed move toward the use of two speakers so that we can begin to manipulate stimulus intensity in a parametric manner, and thus move toward better examining the spatial and temporal aspects of the observed multisensory gain. Another potential caveat to these findings is the role of attention to the unisensory (visual or auditory) stimuli during this task. A variety of studies have demonstrated a relationship between (multi)sensory processing and attention (Stein et al., 1995; Spence and Driver, 2004; Talsma et al., 2010). In the current design it is difficult to control for the differential allocation of attention to one modality or the other, yet such attentional biases are likely. Nonetheless, the pattern of behavioral response argues against a fixed strategy of attending to one of the stimulus modalities, suggesting that there were some attentional resources deployed toward both modalities. Regardless of the distribution of attention, the presence of multisensory gain suggests that even a stimulus in an unattended modality can modulate performance in the attended modality. Future work may also include reversing the training order, where animals would first complete the auditory training followed by visual training before testing under multisensory conditions. In addition, this study focused on the performance under congruent/paired audiovisual trials; however future studies could examine cognitive flexibility or set shifting by utilizing incongruent audiovisual trials. An interesting variant of this task would be to train animals under only one sensory modality condition (e.g., auditory) and then pair this with a separate irrelevant sensory stimulus (e.g., visual) (Lovelace et al., 2003). Finally, future studies may focus on other sensory domains (e.g., tactile, olfaction) that may be more relevant or salient to mice.

A number of recent behavioral studies characterizing multisensory processing in the rat are highly relevant to these results (Raposo et al., 2012, 2014; Carandini and Churchland, 2013; Sheppard et al., 2013). These studies have demonstrated that behavioral gains can be observed under multisensory conditions similar to those found in larger animal models, and the most recent of these studies have evaluated the underlying circuits that may be crucial for audiovisual integration in the rat (Brett-Green et al., 2003; Komura et al., 2005; Hirokawa et al., 2008, 2011). Therefore, with the foundation established for this behavioral paradigm, we believe numerous future studies could be pursued focused on evaluating and linking mechanistic function with the associated behavior under multisensory conditions in the mouse model. More specifically, we believe that the current work will serve as the springboard for identifying the neurobiological substrates and circuits that support these behavioral effects. Classical studies focused on the deep layers of the superior colliculus (SC) have found this to be a watershed structure for the convergence and integration of information from vision, audition and touch (Meredith and Stein, 1983, 1986a,b; Meredith et al., 1987). Further studies demonstrated that lesions to the deeper (i.e., multisensory) layers of the SC cause not only a diminished neuronal response under multisensory conditions, but also result in a dramatic reduction in the associated behavioral benefits (Burnett et al., 2002, 2004, 2007). Thus, one likely substrate for the multisensory behavioral effects shown here is the SC, given its central role in audiovisual detection and localization (Hirokawa et al., 2011). In addition, work in the cat model has shown that this SC-mediated integration is heavily dependent upon convergent cortical inputs that appear to gate the integrative features of SC neurons (Wallace et al., 1992; Wallace and Stein, 1994; Wilkinson et al., 1996; Jiang et al., 2002). With the use of neurophysiological and neuroimaging methods, similar corticotectal circuits have been described in the rat, highlighting the conservation of a similar circuit system in smaller animal models (Brett-Green et al., 2003; Wallace et al., 2004; Menzel and Barth, 2005; Rodgers et al., 2008; Sanganahalli et al., 2009; Sieben et al., 2013). Specifically the selective deactivation to a higher order cortical region (V2L) resulted in a severe disruption in behavioral performance when responding to audiovisual stimuli (Hirokawa et al., 2008). Of greatest interest to this study, there have been a variety of recent studies focused on determining the underlying brain regions and circuits critical for multisensory processing in the mouse model, although none of these studies have examined the behavioral response to multisensory stimuli (Hunt et al., 2006; Cohen et al., 2011; Laramée et al., 2011; Charbonneau et al., 2012; Olcese et al., 2013; Gogolla et al., 2014; Reig and Silberberg, 2014). Understanding whether such a cortical dependency exists in the mouse model is a focus of future inquiry and therefore possible targets of multisensory input include the SC and the cortical region V2L. These mechanistically driven studies would then take advantage of the utility of the mouse model by using both genetic and optogenetic techniques to evaluate the underlying neural mechanisms of multisensory processing.

Another avenue of future research is to evaluate and further characterize mouse models of disease/disorder with known (multi)sensory processing deficits in the human population. Most importantly, the use of mouse models allows for the application of powerful genetic, pharmacologic and optogenetic tools to questions of mechanistic relevance that are not readily available for larger animal models. Two clinical populations with known (multi)sensory dysfunction are schizophrenia and autism (de Gelder et al., 2003, 2005; Behrendt and Young, 2004; Dakin and Frith, 2005; Iarocci and Mcdonald, 2006; Minshew and Hobson, 2008; de Jong et al., 2009; Grossman et al., 2009; Javitt, 2009; Marco et al., 2011; Cascio et al., 2012; O’Connor, 2012; Martin et al., 2013; Wallace and Stevenson, 2014). Recently, there has been an increased focus on linking these behavioral findings with possible neural correlates to gain a better understanding of the atypical (multi)sensory processing observed in these clinical populations (Russo et al., 2010; Brandwein et al., 2012; Stekelenburg et al., 2013). Numerous genetic mouse models of clinical disorders such as autism and schizophrenia have shown behavioral deficits and altered neural connectivity (Silverman et al., 2010; Provenzano et al., 2012; Hida et al., 2013; Karl, 2013; Lipina and Roder, 2014); however, behavioral studies of multisensory function have not yet been reported in these animals. In fact, a recent study demonstrated multisensory processing differences between wild type mice and mouse models of autism at the neuronal level and showed the potential to ameliorate these effects under pharmacologic manipulations (Gogolla et al., 2014). This approach has enormous potential to reveal mechanistic contributions of altered multisensory function to these disease states. The use of our behavioral paradigm, along with pharmacologic or optogenetic techniques, could then allow for the assessment of novel therapeutic approaches that may link altered neural mechanisms to the resultant atypical behavior. Finally, these types of studies would offer great promise as a translational bridge that seeks to better link genetic, phenotypic and neural factors in an effort to better elucidate the contributing role of alterations in sensory function in developmental disorders such as autism or schizophrenia.

Overall, this study has shown that multisensory processing is conserved in the mouse model by demonstrating similar behavioral benefits to those observed throughout numerous larger animal models. With the design of the first behavioral paradigm to assess multisensory function in the mouse, we believe this allows for a whole host of future research opportunities. This type of behavioral task will allow for a variety of mechanistically driven studies focused on the neural underpinnings of multisensory processing, in addition to studies dedicated to evaluating these circuits in models of clinical disorders with known (multi)sensory impairments.

## Conflict of interest statement

The authors declare that the research was conducted in the absence of any commercial or financial relationships that could be construed as a potential conflict of interest.
